# Morphology is not a reliable taxonomic tool for the genus *Lernaea*: molecular data and experimental infection reveal that *L. cyprinacea* and *L. cruciata* are conspecific

**DOI:** 10.1186/s13071-019-3831-y

**Published:** 2019-12-11

**Authors:** Cong J. Hua, Dong Zhang, Hong Zou, Ming Li, Ivan Jakovlić, Shan G. Wu, Gui T. Wang, Wen X. Li

**Affiliations:** 1 Key Laboratory of Aquaculture Disease Control, Ministry of Agriculture, and State Key Laboratory of Freshwater Ecology and Biotechnology, Institute of Hydrobiology, Chinese Academy of Sciences, Wuhan, 430072 People’s Republic of China; 2 Wuhan Institute of Biomedical Sciences, School of Medicine, Jianghan University, Wuhan, 430056 People’s Republic of China; 3 University of Chinese Academy of Sciences, Beijing, 100049 People’s Republic of China; 4 Bio-Transduction Lab, Wuhan, 430075 People’s Republic of China

**Keywords:** *Lernaea*, Parasitic copepods, Phenotypic plasticity, Molecular taxonomy

## Abstract

**Background:**

Species belonging to the genus *Lernaea* are cosmopolitan parasites that can infect many different freshwater fish hosts. Due to a high degree of morphological intraspecific variability and high levels of interspecific similarities, their classification is extremely difficult and controversial. Although the suitability of the shape of cephalic horns has been questioned decades ago by some experimental infection studies, this character still plays the central role in the identification of *Lernaea* spp.

**Methods:**

We used the nominal species *Lernaea cyprinacea* and *Lernaea cruciata* to test the hypothesis that the shape of the anchor can exhibit host-induced morphological variability, and that the two taxa may be synonymous.

**Results:**

We examined 517 wild or farmed specimens of five host fish species (four cyprinids and a mosquitofish), and found that all 16 parasite specimens collected from mosquitofish could be morphologically identified as *L. cruciata*, whereas the remaining 25 parasite specimens were all identified as *L. cyprinacea*. We experimentally infected goldfish and mosquitofish specimens with offspring (copepodids) of a single *L. cyprinacea* specimen: the adult parasites from goldfish were morphologically identified as *L. cyprinacea*, and those from mosquitofish as *L. cruciata*. We then used molecular data to corroborate that all these specimens are conspecific.

**Conclusions:**

Our results suggest that *L. cyprinacea* and *L. cruciata* may be synonyms, misidentified as different species as a result of host-induced morphological variation. Given the current shortage of molecular data for the genus *Lernaea*, in order to resolve the taxonomy of this genus (determine the exact number of species), future studies should aim to sequence as much molecular data as possible, and conduct further experimental infections.

## Background

Cosmopolitan parasitic copepods belonging to the genus *Lernaea* Linnaeus, 1758 (Cyclopoida: Lernaeidae) can infect many different freshwater fish species [1–3], causing lernaeosis, a disease that can cause serious pathogenic effects on their hosts. The taxonomy of this genus is still largely unresolved due to the existence of an exceptionally large number (109) of recorded nominal species [4], a small number of morphological traits useful for species identification, a high degree of intraspecific morphological variability, and a lack of clear morphological distinction between some species [5–8]. As a result, almost half (48) of the nominal species are believed to be synonymous [4].

The shape of the cephalic anchors, also known as the “horns” or “antlers”, and their processes, has been traditionally used as the most reliable characteristics for taxonomic identification of *Lernaea* spp. [9, 10]. However, experimental infection studies put a major question mark over the validity of the prevailing taxonomy of the genus by showing that the anchor exhibits high intraspecific variation [5, 11]. For example, Harding [6] and Fryer [7] have shown that the growth and orientation of the anchor are affected by the anatomy of the host. As discussed by Fryer [7], Yashouv [12] collected larvae from adults settled on one host and then successfully infected another fish species (host not specified by Fryer) and tadpoles with these larvae; most of the specimens from carp and buffalo fish were assignable to *L. cyprinace*a, but specimens from *Gambusia* were morphologically notably different from *L. cyprinacea*. A subsequent study of Poddubnaya (1973) even further casted the shadow of doubt over the usefulness of the anchor for species identification: when different hosts were infected by larvae from a single batch of *L. elegans* (a synonym of *L. cyprinacea*) eggs, adult parasites exhibited different anchor characteristics, some of which corresponded to other described species of *Lernaea* [13]. Finally, a recent study [10], used *18S* and *28S* gene fragments to identify four *Lernaea* specimens, which could be assigned to different species on the basis of their morphology (the authors did not indicate which species), and found that the specimens shared a similar genotype, so all were identified as *L. cyprinacea*. These results clearly demonstrate that the anchor characteristics are often merely structural adaptations of a single parasitic specimen to a different host species, and therefore have very limited reliability for taxonomic purposes.

Although molecular data (partial sequences of *18S* and *28S* rRNA) have been used in the identification of *Lernaea* species [10, 11, 14–16], their availability remains extremely limited. To illustrate this, in December 2018 there were only 21 sequences for just two genes (*18S* and *28S*), and a mitochondrial genome sequence, all belonging to a single species, *L. cyprinacea*, available in the GenBank database (Additional file 1: Table S1).

The reliability of the two single gene-based molecular markers (*18S* and *28S*) that have been traditionally used in the identification and phylogenetic studies of *Lernaea* species remains questionable, or at least unconfirmed, largely due to a limited number of studies and limited amount of molecular data publicly available [10, 16]. The DNA barcode marker, *cox*1, has not been used in the identification or phylogenetic studies. Therefore, we can conclude that due to the shortage of molecular data and the shortcomings of the anchor as a tool for taxonomy and identification, scientists currently do not have a single sufficiently reliable tool for the identification of *Lernaea* species at disposal.

*Lernaea cruciata* Lesueur, 1824, first reported from the body surface of the rock bass *Cichla aenea* [17], has been subsequently reported from more than a dozen fish species [18]. The anchor of this species is X-shaped, with four simple and short arms approximately equal in size. *Lernaea cyprinacea* is the most widely distributed species of the genus [19], which also exhibits very low host specificity, infecting a wide range of freshwater fishes, as well as some amphibians [19, 20], but its anchoring apparatus is much more complex. It also has two pairs of arms [21], but the dorsal pair is larger than the ventral pair, it branches out into pairs of processes at the tip; hence, some studies refer to it as the T- or Y-shaped dorsal ramified pair, or as “antlers” [5, 21–23]. For instance, experimental infection trials on *Gambusia* sp. infected with *L. cyprinacea* showed a significant change in the shape of the anchor. In this case, the anchor of *L. cyprinacea* specimens developed into the adults which were similar to those described for *L. gobioptera* [24] and/or *L. cruciata* [17, 25], and not the typical form of *L. cyprinacea*. However, despite the fact that the evidence for its extreme variability was presented several decades ago, perhaps due to the absence of other morphological features that could be used for this purpose, the shape of the anchor remains commonly used as the most useful morphological trait, for the identification of *Lernaea* species [10, 11, 16, 26–29]. The issue of reliability of this trait has not been revisited in decades, and it has never been studied using a combination of experimental infection and molecular tools. Our working hypothesis was that *L. cyprinacea* and *L. cruciata* are synonyms, but the shape of the anchor is prone to host-induced morphological variability, which results in taxonomic misidentification. This would also prove that this morphological trait is not a reliable tool for the identification of (at least these two) *Lernaea* species. In a preliminary survey, specimens morphologically (anchor) corresponding to *L. cyprinacea* were found on four cyprinid fish species, whereas specimens morphologically corresponding to *L. cruciata* were found only on *Gambusia holbrooki* (the eastern mosquitofish, referred to as mosquitofish henceforth). To test our hypothesis, and the taxonomic validity of *L. cruciata*, we infected different host species with these specimens, and used molecular data to corroborate the identity of specimens collected from different hosts.

## Methods

### Sample collection and identification of *Lernaea cyprinacea* and *L. cruciata* from wild fishes

In 2016 and 2017, we collected 517 fish specimens belonging to 5 species (*Carassius auratus*, *Cyprinus carpio*, *Erythroculter ilishaeformis*, *Gambusia holbrooki*, *Squaliobarbus curriculus*) from six locations, comprising wild, farmed and captive fish populations (Table 1). Except for the mosquitofish, which were captured using dip nets with 5 mm stretched mesh size, fishes were captured using trawl nets. Immediately after sampling, the fish specimens were visually examined (oral cavity, branchial cavities, head, fins and skin) for the presence of copepod parasites. Uninfected fish were returned to the water unharmed, and fishes infected with copepod parasites were placed in aerated tanks and transported to the laboratory alive. Parasites were collected from hosts using forceps and a dissecting needle, and preserved in 70% ethanol for detailed identification by light microscopy. Species identification was conducted according to the available literature [16, 17, 25, 30, 31].

**Table 1:** List of collected *Lernaea* specimens with collection details

Morphological identification	*n*	Host fish	Location	Province	Collection date
Specimens in wild-caught fishes					
*L. cyprinacea*	6	*Carassius auratus* (*n* = 60)	Tangxun Lake (30°24′N, 114°23′E)	Hubei	15 May 2016
	13	*Carassius. auratus* (*n* = 58)	Taibai Lake (29°58′N, 115°50′E)	Hubei	15 Dec 2016
	3	*Squaliobarbus curriculus* (*n* = 20)	Taibai Lake (29°58′N, 115°50′E)	Hubei	15 Dec 2016
	1	*Erythroculter ilishaeformis* (*n* = 7)	Yangluo Farm (30°48′N, 114°36′E)	Hubei	3 Mar 2017
	2	*Cyprinus carpio* (*n* = 4)	Donghu Lake (30°33′N, 114°21′E)	Hubei	20 Mar 2016
*L. cruciata*	13	*Gambusia holbrooki* (*n* = 210)	Shaoguan (24°47′N, 113°35′E)	Guangdong	6 Oct 2016
	3	*Gambusia holbrooki* (*n* = 158)	Hengyang (26°54′N, 112°36′E)	Hunan	11 Jun 2017
Species from laboratory infections					
*L. cyprinacea*	15	*Carassius auratus* (*n* = 15)		Hubei	1 May 2017
*L. cruciata*	5	*Gambusia holbrooki* (*n* = 15)		Hubei	1 May 2017

*Abbreviation*: n, number of collected *Lernaea* specimens

### Experimental infections of goldfish and mosquitofish with *L. cyprinacea*

Goldfish (*Carassius auratus*) specimens with a mean body weight of 6.24 ± 0.78 g were obtained from a local fish farm in Wuhan City, China. The population had no history of infection with *L. cyprinacea* (i.e. no previous reports of visible signs of infection). Mosquitofish were collected from the Donghu Lake (Table 1), and (apparently) gravid female specimens were selected to be taken to the laboratory and used to produce broodstock *via* induced breeding (mosquitofish is ovoviviparous). The fingerlings used for the experimental infection were reared under controlled nursery conditions; they were apparently healthy and of same age. The two experimental populations were kept in two separate, aerated tanks (30% water exchanged fortnightly) for 30 days: goldfish (*n* = 125) in a 500 litre tank, and mosquitofish (*n* =73) in a 30 litre tank. To remove all ectoparasites, all experimental fish were treated with three consecutive baths in 1:10,000 formalin solution for 12 h at 48 h intervals [32]. Treated fish were then examined under a dissection microscope to ensure that they are free of *Lernaea* parasites. A subset of specimens of both species (*n* = 30 for each species) was then selected for the experimental inspection. These specimens were moved to small (5 litre) plastic aquaria (1 specimen/aquarium) filled with static dechlorinated tap water and equipped with aerators and acclimatized and quarantined for at least seven days before being used in experiments. The light:dark cycle was 12:12 h, water pH ranged between 7.4–8.0, and temperature ranged between 24–28 °C. Fish were fed twice daily with commercial pelleted feed, at 2% of the estimated total fish biomass. Egg-sacs of a *Lernaea* specimen, obtained from *Erythroculter ilishaeformis* obtained from the Yangluo farm (Table 1), morphologically and genetically (*cox*1, *18S* and *28S*; GenBank accession numbers MH982220, MH982197 and MH982208, respectively) identified as *L. cyprinacea*, were removed and cultured under laboratory conditions as previously described [33]. This laboratory stock of *L. cyprinacea* (offspring of a single parasite) used for all experimental infestations was maintained on a population (*n* = 20) of laboratory-reared goldfish. Copepodids-I (the infective stages) of *L. cyprinacea* were obtained by culturing parasite eggs extracted from the egg-sacs of gravid female parasites under controlled laboratory conditions. Viability of the hatched copepodid-I specimens was inferred by counting the active swimming specimens in diluted stock suspensions under the microscope (4× magnification). Only active parasites were taken into consideration for calculating the number of copepodid-I specimens used to infect each group.

Fifteen goldfish and 15 mosquitofish specimens were isolated and exposed to infective stages of *L. cyprinacea* at the rates of 70 and 30 copepodids, respectively. Each fish specimen remained isolated in a container with 5 litres of water to prevent the other fish from removing their parasites, and examined daily using a stereomicroscope to determine the success of the infection. Two parallel control groups (same species, same size, *n* =15), unexposed to parasites, were maintained in an identical environment (isolated in aquaria).

Water exchange and removal of faecal material were not carried out during the initial seven days post-infection to prevent accidental removal of free-living infective stages. After this initial period, faecal and uneaten feed material settled at the bottom of the pools were removed by siphoning with plastic tubes regularly. Regular water exchange (25%) was performed on alternate days. Observations were conducted daily from the onset of infection. Parasites with egg-sacs were removed when they were fully developed. *Lernaea* parasites were carefully detached using forceps and dissecting needle, and examined immediately. Species identification was conducted as described above.

### Morphological and statistical analysis

**Fig. 1:**
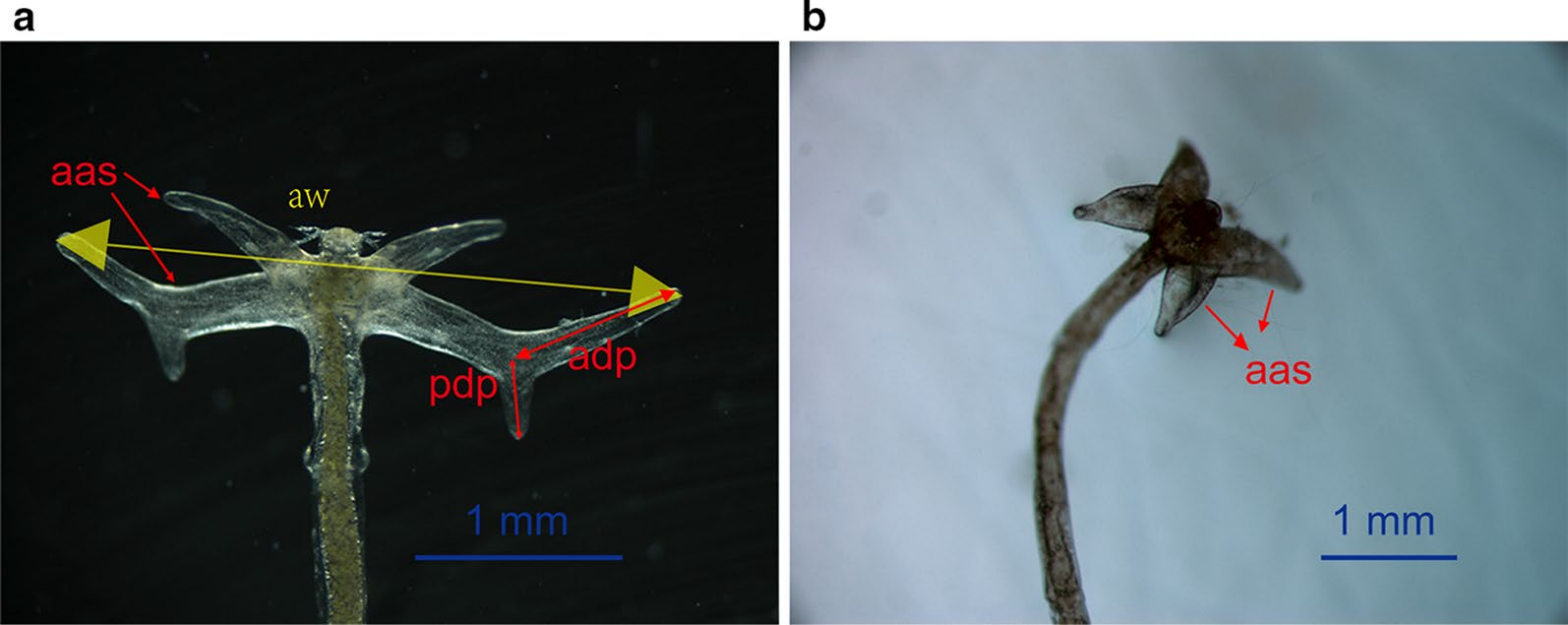
**a** A representative *Lernaea cyprinacea* specimen from goldfish. **b** A representative *Lernaea cruciata* specimen from mosquitofish. *Abbreviations*: aas, anchor arms; aw, anchor width; va, ventral arm; adp, anterior dorsal process; pdp, posterior dorsal process

All of the examined parasite specimens were photographed, and subjected to detailed morphological and morphometric analyses. The specimens were visualized and photographed under a stereomicroscope (Leica S8AP0, Wetzlar, Germany), equipped with a SPOT Insight 2.0 Mp digital camera (Sterling Heights, MI, USA). The length of the whole body, arms and processes, and anchor width were measured in mm (Fig. 1), with allowance being made for the bends and curves of the body, as described by Robinson & Avenant-Oldewage [28].

Principal components analysis (PCA) was performed to determine the difference between different groups (i.e. *L. cyprinacea* and *L. cruciata* samples collected in the wild and from experimental infections). All physical variables were standardized to zero mean and unit variance to make them dimensionless. Results were considered significant at the 95% level (*P* < 0.05). Ordination and multivariate analysis of variance (MANOVA) analyses were performed using the *vegan* package in R version 3.6.1 [34, 35].

### Molecular data amplification and sequencing

Molecular analysis was performed for 7 *L. cyprinacea* and 5 *L. cruciata* specimens collected from host fish in the wild and from those collected during the laboratory infection experiment. Total genomic DNA was extracted from the posterior part of the parasite body (to preserve the anchor intact) using TIANamp Micro DNA Kit (Tiangen Biotech, Beijing, China), according to the manufacturer’s instructions. DNA was stored at − 20 °C for subsequent molecular analysis. Three molecular markers were amplified by PCR and sequenced: a fragment of the mitochondrial cytochrome *c* oxidase subunit 1 (*cox*1), a partial sequence of the *18S* rRNA gene, and a partial sequence of the *28S* rRNA gene. *cox*1 was amplified using newly designed primers (forward: 5′-TAG TTG GAA TTT GGG CTG GC-3v and reverse: 5′-ATT AGG GGC CTT GTT GGG AAG-3′). The PCR reaction mix (20 µl) was comprised of 1 µl genomic DNA, 0.6 µl of both primers, 7.4 µl ddH2O, 10 µl 2× PCR buffer (Mg2+, dNTP plus, Takara, Dalian, China), 0.4 µl rTaq polymerase (250 U, Takara); conditions were: initial denaturation at 98 °C for 2 min, 40 cycles of 98 °C for 10 s, 50 °C for 15 s, 68 °C for 1 min/kb, and the final extension at 68 °C for 10 min. Primers and PCR parameters for *18S* and *28S* were previously published [14]. Amplicons were subjected to electrophoresis on a 1.2% agarose gel stained with GoldView dye (Solarbio, Beijing, China), and then purified and sequenced by the Sangon Co. (Shanghai, China). The 36 newly generated sequences were compared with similar sequences available in the GenBank database using the Basic Local Alignment Search Tool (BLAST) [36] to confirm their identity and deposited in GenBank under the accession numbers MH982192-MH982227 (Additional file 2: Table S2).

### Comparative and phylogenetic analyses

Sequences obtained in this study (*cox*1, *18S*, *28S* genes of the nominal *L. cyprinacea* and *L. cruciata* specimens) were aligned with all *Lernaea* spp. (all belonging to *L. cyprinacea*) available on GenBank (Additional file 1: Table S1) using MAFFT 7.149 [37]. For comparative analyses, *18S* and *28S* sequences obtained from GenBank that did not exhibit a full overlap with the sequences obtained in this study were removed from the alignment. Multiple alignment, pairwise identity and the number of variable sites among the sequences of *cox*1, *18S* and *28S* were conducted using Geneious 8.1.3 [38]. The number of haplotypes for each molecular marker was calculated using DnaSP v6.11.01 [39]. Sites with gaps and missing data were not considered.

For phylogenetic analyses, we used all available *Lernaea* sequences and added outgroups. As the only *cox*1 gene sequence belonging to the family Lernaeidae available on GenBank was that from the mitogenome of *L. cyprinacea* (KM235194), for the outgroup we used two sequences for *Sinergasilus polycolpus* belonging to another family of the order Cyclopoida (Ergasilidae) (Additional file 3: Table S3). For the *18S* data, we selected all available homologues belonging to the closest-related [14] genus in the family Lernaeidae, *Lamproglena*, to root the tree (Additional file 3: Table S3). For the *28S* dataset, five sequences belonging to two *Lamproglena* spp. were chosen as the outgroup (Additional file 3: Table S3). For the fourth dataset, to maximize the phylogenetic resolution, we concatenated these three genes into a single alignment (*cox*1, *18*S and *28S*). Due to lack of suitable data, we did not include an outgroup in this analysis. Phylogenetic analyses were conducted using four datasets (*cox*1, *18*S, *28S* and concatenated) and two methods: maximum likelihood (ML) and Bayesian inference (BI). ML was conducted using IQTree v1.6.3 [40], and BI using MrBayes 3.2 [41]. We used the akaikeʼs information criterion (AIC), implemented in ModelFinder [42] in IQ-Tree, to select the best-fit evolutionary models for each dataset (Additional file 4: Table S4).

## Results

### Morphology of *Lernaea* spp. collected in the wild

After carefully inspecting 517 fish specimens belonging to five species (Table 1) collected in the wild, and discarding all parasite specimens which could not be identified with confidence (not fully developed or with incomplete anchor arms, usually damaged while removing from the body surface of fish), we morphologically identified 25 specimens as *L. cyprinacea* and 16 as *L. cruciata.* Whereas all *L. cruciata* specimens were collected from the mosquitofish, *L. cyprinacea* specimens were collected from hosts belonging to four different species: *Cyprinus carpio*, *C. auratus*, *Squaliobarbus curriculus* and *Culter alburnus*.

### Experimental infection results and comparative morphology of *L. cyprinacea* and *L. cruciata*

**Fig. 2:**
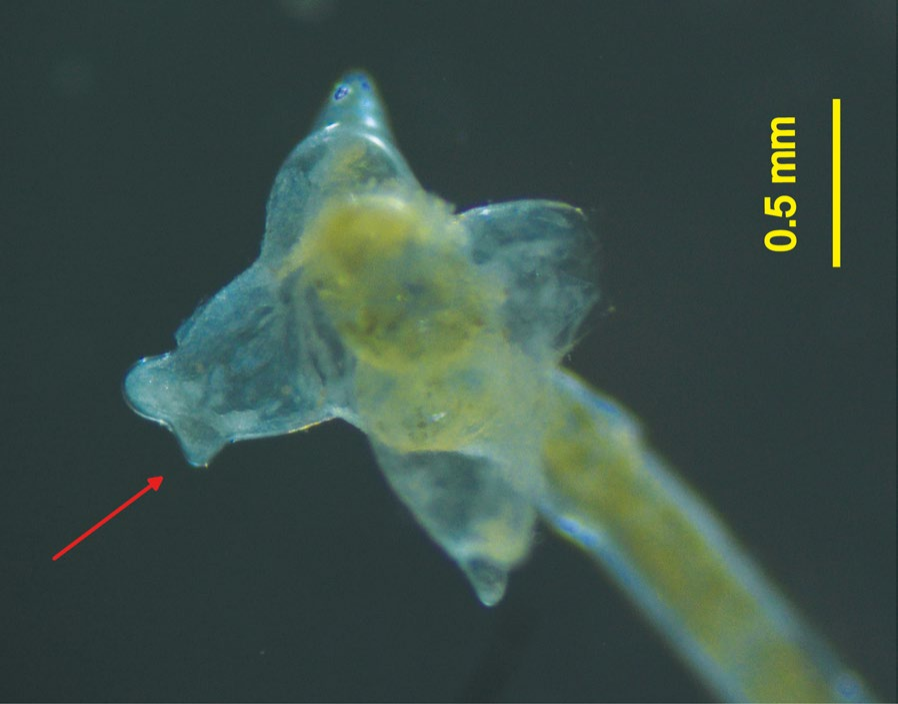
A *Lernaea* specimen collected from an experimentally infected mosquitofish, and identified as *L. cruciata*, exhibiting minuscule processes on one of its arms, somewhat resembling those of *L. cyprinacea*. The arrow highlights the feature somewhat resembling a posterior dorsal process

Specimens found on fish after the experimental infection were regarded as mature (fully developed) when they were ovigerous. In the *C. auratus* infection experiment, 15 *Lernaea* specimens were collected from six fish specimens and morphologically identified as *L. cyprinacea*. In the mosquitofish infection experiment, only 5 adult *Lernaea* specimens were collected from 5 fish and all morphologically identified as *L. cruciata*. One of these five parasites exhibited somewhat mixed morphological traits, with one of its arms exhibiting minuscule processes, but it predominantly exhibited a morphology corresponding to *L. cruciata* (Fig. 2).

Among the parasites obtained in experimental infection, the average body length and anchor width of *L. cyprinacea* (8.86 ± 1.71 and 2.31 ± 1.37 mm, respectively) were greater than those of *L. cruciata* specimens (7.10 ± 1.00 and 1.19 ± 0.29 mm, respectively) (Table 2). The average ventral anchor arms of *L. cyprinacea* (0.55 ± 0.21 mm) were also longer than those of *L. cruciata* (0.49 ± 0.10 mm). However, none of these differences between samples of *L. cyprinacea* and *L. cruciata* were statistically significant (t-test: body length, *t*_(18)_ = 2.159, *P* = 0.45, anchor width, *t*_(18)_ = 1.78, *P* = 0.92, ventral arms, *t*_(18)_ = 0.654, *P* = 0.522).

**Table 2:** Morphometrics of *Lernaea cyprinacea* (Lcy) and *L. cruciata* (Lcr) specimens collected from experimental infections and in the wild

Sample	Character	Mean ± SD	Range	CV (%)
Lcy wild	Body length	7.79 ± 2.44	5.01–12.40	31.35
	Anchor width	3.31 ± 1.09	1.45–6.03	32.91
	Anterior dorsal processes	0.80 ± 0.33	0.07–1.73	40.73
	Posterior dorsal processes	0.42 ± 0.19	0.15–0.95	45.68
	Ventral arms	0.65 ± 0.23	0.26–1.13	34.50
Lcr wild	Body length	7.11 ± 0.92	5.69–8.26	12.98
	Anchor width	1.16 ± 0.34	0.71–2.00	29.11
	Dorsal processes	0.56 ± 0.18	0.31–1.00	33.07
	Ventral arms	0.53 ± 0.16	0.33–1.00	30.82
Lcy lab	Body length	8.86 ± 1.71	6.67–12.3	19.30
	Anchor width	2.31 ± 1.37	0.87–6.13	59.24
	Anterior dorsal processes	0.90 ± 0.39	0.26–1.74	42.91
	Posterior dorsal processes	0.44 ± 0.20	0.21–0.96	44.82
	Ventral arms	0.55 ± 0.21	0.31–1.01	37.39
Lcr lab	Body length	7.10 ± 1.00	5.79–8.05	14.10
	Anchor width	1.19 ± 0.29	0.89–1.64	24.22
	Dorsal processes	0.48 ± 0.09	0.33–0.56	19.43
	Ventral arms	0.49 ± 0.10	0.34–0.58	20.15

*Note*: Pairwise comparison *P-*values are available in Table 2

*Abbreviations*: SD, standard deviation; CV, coefficient of variation; lab, experimental infections

### Multivariate morphometric comparison of the wild and experimental *L. cyprinacea* and *L. cruciata* specimens

**Fig. 3:**
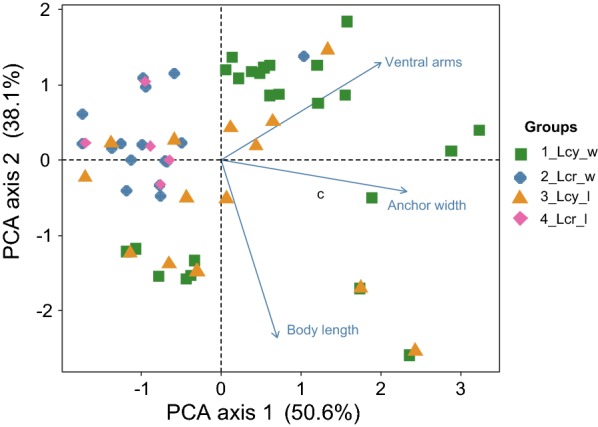
Principal components analysis (PCA) ordination showing the difference of morphological features between the two different *Lernaea* species collected in the wild and from an experimental (laboratory) infection. *Abbreviations*: Lcy, *L. cyprinacea*; Lcr, *L. cruciate*; l, specimens collected from the laboratory infection; w, specimens collected in the wild. *Notes*: Samples are divided into four groups (1 to 4). For example, 4_Lcr_l indicates group 4, comprising specimens of *L. cruciata* collected from the laboratory infection

PCA (Fig. 3) revealed that PC1, mainly determined by the width of the anchor and the length of the ventral arms, explained 50.6% of the variation contained within all morphological characteristics. PC2, mainly associated with the length of the body and ventral arms, explained 38.1% of the total variation. Therefore, PC1 and PC2 combined explained almost 90% (88.7%) of the variance contained within the dataset. Apart from a single outlier (Fig. 3), *L. cruciata* specimens collected in the wild and from the laboratory infection clustered together. The distribution of *L. cyprinacea* specimens (both wild and laboratory-reared) was scattered, but several laboratory-reared specimens overlapped with the *L. cruciata* cluster.

### Sequence comparisons

Partial *cox*1, *18S* and *28S* genes (1241, 1389 and 706 bp, respectively) all exhibited very high identity (BLAST) values to the corresponding available *L. cyprinacea* sequences in the GenBank database: 99%, 99–100% and 99–100%, respectively (Additional file 5: Table S5). We aligned our sequences with all fully overlapping *L. cyprinacea* homologs available in the GenBank.

#### *cox*1

All *cox*1 sequences obtained in this study and one *cox*1 sequence of *L. cyprinacea* downloaded from GenBank were used to compare the sequence similarity. High pairwise identity (98.2–100%) was found among the *cox*1 sequences of *L. cyprinacea* (Lcy) and putative *L. cruciata* (Lcr) (Table 3). The highest number of variable sites among the sequences was 22 (between Lcy2 and Lcy6, Lcy2 and Lcy7). There were 9 haplotypes and 45 variable sites among these sequences.

**Table 3:** Pairwise identity (below the diagonal) and variable sites (above the diagonal) for the *cox*1 dataset

Sequence		1	2	3	4	5	6	7	8	9	10	11	12	13
1	Lcy1_w		15	13	2	8	19	19	18	18	18	15	15	14
2	Lcy2_w	98.8		4	14	15	22	22	21	21	21	14	14	13
3	Lcy3_w	99	99.7		12	13	20	20	19	19	19	12	12	11
4	Lcy4_w	99.8	98.9	99		8	19	19	18	18	18	15	15	14
5	Lcy5_w	99.4	98.8	99	99.4		19	19	18	18	18	15	15	14
6	Lcy6_l	98.5	98.2	98.4	98.5	98.5		0	5	5	5	20	20	17
7	Lcy7_l	98.5	98.2	98.4	98.5	98.5	100		5	5	5	20	20	17
8	Lcr1_l	98.5	98.3	98.5	98.5	98.5	99.6	99.6		0	0	19	19	16
9	Lcr2_l	98.5	98.3	98.5	98.5	98.5	99.6	99.6	100		0	19	19	16
10	Lcr4_w	98.5	98.3	98.5	98.5	98.5	99.6	99.6	100	100		19	19	16
11	Lcr5_w	98.8	98.9	99	98.8	98.8	98.4	98.4	98.5	98.5	98.5		0	13
12	Lcr6_w	98.8	98.9	99	98.8	98.8	98.4	98.4	98.5	98.5	98.5	100		13
13	LcyGB	98.9	99	99.1	98.9	98.9	98.6	98.6	98.7	98.7	98.7	99	99	

*Notes*: Lower left half shows pairwise identity (the % of bases/residues that are identical), and upper right half shows the number of variable sites. Sequences 1–12 belong to specimens from this study, and number 13 (LcyGB) is from GenBank (*L. cyprinacea cox*1, KM235194)

*Abbreviations*: Lcy, *Lernaea cyprinacea*; Lcr: *L. cruciate*; w, specimen collected in the wild; l, specimen collected from the experimental (laboratory) infection

## 18S

Twelve *18S* sequences of *L. cyprinacea* (seven sequenced in this study and five from GenBank) and five *18S* sequences of *L. cruciata* (sequenced in this study) also exhibited very high pairwise identity (99.8–100%) (Table 4). There were 7 haplotypes and only 7 variable sites. It is also noteworthy that all of the newly generated *18S* sequences of *L. cyprinacea*, two *18S* sequences of *L. cyprinacea* downloaded from GenBank (DQ107555, KP235363), and two *18S* sequences of *L. cruciata*, shared the same haplotype.

**Table 4:** Pairwise identity (below the diagonal) and variable sites (above the diagonal) for the *18S* dataset

Sequence		1	2	3	4	5	6	7	8	9	10	11	12	13	14	15	16	17
1	Lcy1_w		1	0	0	0	0	0	0	0	1	1	2	2	2	2	1	0
2	Lcy2_w	99.9		1	1	1	1	1	1	1	2	2	3	3	2	3	2	1
3	Lcy3_w	100	99.9		0	0	0	0	0	0	1	1	2	2	2	2	1	0
4	Lcy4_w	100	99.9	100		0	0	0	0	0	1	1	2	2	2	2	1	0
5	Lcy5_w	100	99.9	100	100		0	0	0	0	1	1	2	2	2	2	1	0
6	Lcy6_l	100	99.9	100	100	100		0	0	0	1	1	2	2	2	2	1	0
7	Lcy7_l	100	99.9	100	100	100	100		0	0	1	1	2	2	2	2	1	0
8	Lcr1_l	100	99.9	100	100	100	100	100		0	1	1	2	2	2	2	1	0
9	Lcr2_l	100	99.9	100	100	100	100	100	100		1	1	2	2	2	2	1	0
10	Lcr4_w	99.9	99.9	99.9	99.9	99.9	99.9	99.9	99.9	99.9		2	3	3	3	3	2	1
11	Lcr5_w	99.9	99.9	99.9	99.9	99.9	99.9	99.9	99.9	99.9	99.9		3	3	3	3	2	1
12	Lcr6_w	99.9	99.8	99.9	99.9	99.9	99.9	99.9	99.9	99.9	99.8	99.8		4	4	4	3	2
13	LcyGB	99.9	99.8	99.9	99.9	99.9	99.9	99.9	99.9	99.9	99.8	99.8	99.7		2	2	3	2
14	LcyGB	99.9	99.9	99.9	99.9	99.9	99.9	99.9	99.9	99.9	99.8	99.8	99.7	99.9		2	3	2
15	LcyGB	99.9	99.8	99.9	99.9	99.9	99.9	99.9	99.9	99.9	99.8	99.8	99.7	99.9	99.9		3	2
16	LcyGB	99.9	99.9	99.9	99.9	99.9	99.9	99.9	99.9	99.9	99.9	99.9	99.8	99.8	99.8	99.8		1
17	LcyGB	100	99.9	100	100	100	100	100	100	100	99.9	99.9	99.9	99.9	99.9	99.9	99.9	

*Notes*: Lower left half shows pairwise identity (the % of bases/residues that are identical), and upper right half shows the number of variable sites. Sequences 1–12 belong to specimens from this study, and numbers 13–17 (LcyGB) are from GenBank: DQ107554-DQ107557 and KP235363, respectively

*Abbreviations*: Lcy, *Lernaea cyprinacea*; Lcr, *L. cruciate*; w, specimen collected in the wild; l, specimen collected from the laboratory infection

## 28S

Twelve *28S* sequences of *L. cyprinacea* (seven sequenced in this study and four downloaded from GenBank) and five *28S* sequences of *L. cruciata* (all sequenced in this study) were compared (Table 5). Due to the poorly conserved 3′-end of the LcyGB KM281817 sequence (No. 17), which might be a sequencing artefact, pairwise identity between this sequence and other sequences (98.9–99.1%) was marginally lower than pairwise identity among all remaining sequences (99.4–100%). Variable sites between KM281817 and other sequences ranged from 6 to 8, while variable sites among the remaining sequences ranged from 0 to 4. There were 10 haplotypes and 15 variable sites among all sequences. It is also noteworthy that three *28S* sequences of *L. cyprinacea* (Lcy1, Lcy3, Lcy6) and all *28S* sequences of *L. cruciata* shared the same haplotype.

**Table 5:** Pairwise identity (below the diagonal) and variable sites (above the diagonal) for the *28S* dataset

Sequence		1	2	3	4	5	6	7	8	9	10	11	12	13	14	15	16	17
1	Lcy1_w		1	0	2	2	0	1	0	0	0	0	0	1	2	1	1	6
2	Lcy2_w	99.9		1	3	3	1	2	1	1	1	1	1	2	3	2	2	7
3	Lcy3_w	100	99.9		2	2	0	1	0	0	0	0	0	1	2	1	1	6
4	Lcy4_w	99.7	99.6	99.7		4	2	3	2	2	2	2	2	3	4	3	3	8
5	Lcy5_w	99.7	99.6	99.7	99.4		2	3	2	2	2	2	2	3	4	3	3	8
6	Lcy6_l	100	99.9	100	99.7	99.7		1	0	0	0	0	0	1	2	1	1	6
7	Lcy7_l	99.9	99.7	99.9	99.6	99.6	99.9		1	1	1	1	1	2	3	2	2	7
8	Lcr1_l	100	99.9	100	99.7	99.7	100	99.9		0	0	0	0	1	2	1	1	6
9	Lcr2_l	100	99.9	100	99.7	99.7	100	99.9	100		0	0	0	1	2	1	1	6
10	Lcr4_w	100	99.9	100	99.7	99.7	100	99.9	100	100		0	0	1	2	1	1	6
11	Lcr5_w	100	99.9	100	99.7	99.7	100	99.9	100	100	100		0	1	2	1	1	6
12	Lcr6_w	100	99.9	100	99.7	99.7	100	99.9	100	100	100	100		1	2	1	1	6
13	LcyGB	99.9	99.7	99.9	99.6	99.6	99.9	99.7	99.9	99.9	99.9	99.9	99.9		1	2	2	7
14	LcyGB	99.7	99.6	99.7	99.4	99.4	99.7	99.6	99.7	99.7	99.7	99.7	99.7	99.9		3	3	8
15	LcyGB	99.9	99.7	99.9	99.6	99.6	99.9	99.7	99.9	99.9	99.9	99.9	99.9	99.7	99.6		2	7
16	LcyGB	99.9	99.7	99.9	99.6	99.6	99.9	99.7	99.9	99.9	99.9	99.9	99.9	99.7	99.6	99.7		7
17	LcyGB	99.1	99	99.1	98.9	98.9	99.1	99	99.1	99.1	99.1	99.1	99.1	99	98.9	99	99	

*Notes*: Lower left half shows pairwise identity (the % of bases/residues that are identical), and upper right half shows the number of variable sites. Sequences 1–12 belong to specimens from this study, and numbers 13–17 (LcyGB) are from GenBank: DQ107546, DQ107547, DQ107548, KP235364, KM281817, respectively

*Abbreviations*: Lcy, *Lernaea cyprinacea*; Lcr, *L. cruciate*; w, specimen collected in the wild; l, specimen collected from the laboratory infection

### Phylogenetic analyses

**Fig. 4:**
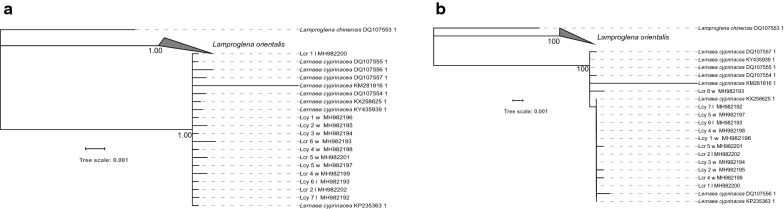
Phylogenetic trees inferred using the *18S* dataset and Bayesian inference (BI) (**a**) and maximum likelihood (ML) (**b**) methods. Numbers next to nodes indicate bootstrap (ML)/posterior probability (BI) support. *Abbreviations*: Lcy, *Lernaea cyprinacea*; Lcr, *L. cruciate*; w, specimens collected in the wild; l, specimens collected from the laboratory infection

**Fig. 5:**
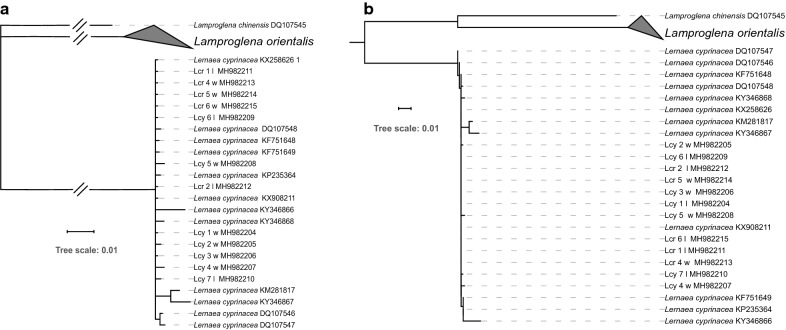
Phylogenetic trees inferred using the *28S* dataset and Bayesian inference (BI) (**a**) and maximum likelihood (ML) (**b**) methods. Numbers next to nodes indicate bootstrap (ML)/posterior probability (BI) support. *Abbreviations*: Lcy, *Lernaea cyprinacea*; Lcr, *L. cruciate*; w, specimens collected in the wild; l, specimens collected from the laboratory infection

**Fig. 6:**
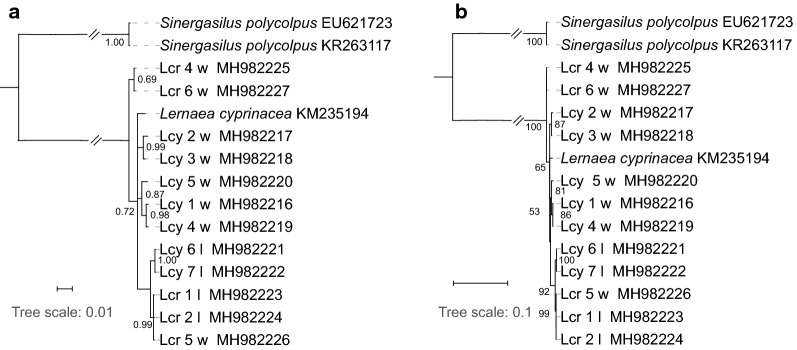
Phylogenetic trees inferred using the *cox*1 dataset and Bayesian inference (BI) (**a**) and maximum likelihood (ML) (**b**) methods. Numbers next to nodes indicate bootstrap (ML)/posterior probability (BI) support. *Abbreviations*: Lcy, *Lernaea cyprinacea*; Lcr, *L. cruciate*; w, specimens collected in the wild; l, specimens collected from the laboratory infection

**Fig. 7:**
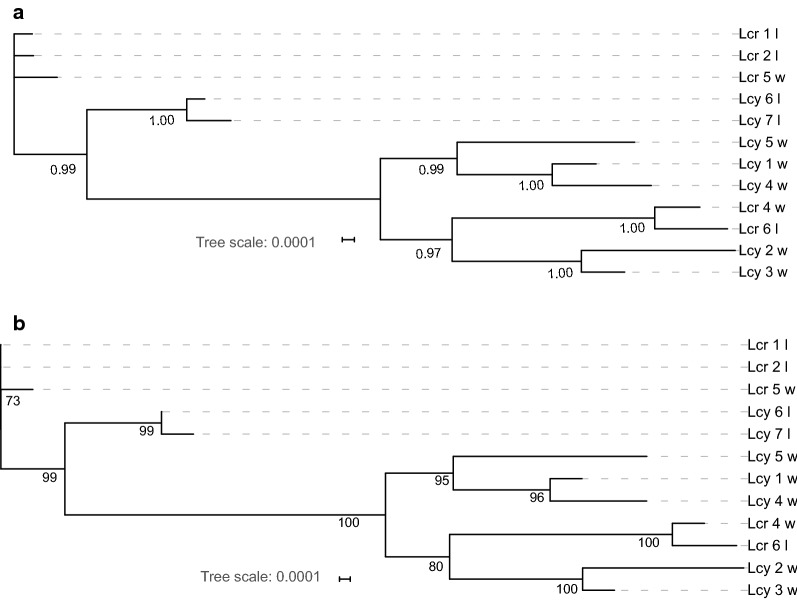
Phylogenetic trees inferred using the concatenated genes (*cox*1, *18S* and *28S*) dataset, and Bayesian inference (BI) (**a**) and maximum likelihood (ML) (**b**) methods. Numbers next to nodes indicate bootstrap (ML)/posterior probability (BI) support. *Abbreviations*: Lcy, *Lernaea cyprinacea*; Lcr, *L. cruciate*; w, specimens collected in the wild; l, specimens collected from the laboratory infection

Despite minor variations in the topology between different algorithms (ML and BI) and datasets (*18S*, *28S*, *cox*1, concatenated genes), all eight obtained phylograms (Figs. 4, 5, 6, 7) produced identical results in two important aspects: monophyletic *Lernaea* clade and highly admixed (paraphyletic) *L. cyprinacea* and *L. cruciata* sequences. Sequences from the two morphospecies did not exhibit clear clustering according to morphotypes or the geographical origin. In contrast, they appeared to be randomly distributed within the cluster.

## Discussion

To date, species of *Lernaea* are still mainly differentiated on the basis of a combination of morphological characters, primarily the shape of the anchors, which is believed to be the most reliable characteristic for identification. However, it has been shown more than half a century ago that anchor morphology to some extent depends on the thickness of the tissue to which the parasite is attached [6, 7]. The age of the parasite, host species, and the site of infestation, can all affect the shape of anchors in *Lernaea* spp. [5, 6]. Most notably, after infecting different fish hosts with larvae from a single batch of eggs of *L. cyprinacea*, Yashouv [12] observed specimens with different anchor shapes, some of which corresponded morphologically to other described *Lernaea* species. However, Yashouv [12] did not have molecular tools at disposal to corroborate this observation. Herein, we compared morphological characteristics of specimens collected in the wild, conducted a laboratory infection experiment wherein different fish hosts were infected with the offspring of a single specimen of *L. cyprinacea*, and sequenced nuclear and mitochondrial genes. Our results showed that the offspring of *L. cyprinacea* collected from mosquitofish could be morphologically identified as *L. cruciata*, while genetically they corresponded to the parental species, *L. cyprinacea*. The observed morphological variation must therefore be host-induced phenotypic variation, affecting the diagnostic morphological features. Despite the lack of sequences for other *Lernaea* species on GenBank, very high identity values among the gene fragments sequenced in this study indicate that specimens from our study genetically correspond to *L. cyprinacea*. Therefore, our results show that the shape of the anchors cannot be considered as a reliable character for the identification of *Lernaea* spp. As there may exist some reproductive barriers between populations parasitizing on different host species, or putatively a genetic preference for a certain host species, further studies should target samples from other populations and a broad range of hosts, and attempt to precisely (if possible) identify the genetic boundaries of this species and the amount of gene flow between its different morphotypes. Importantly, the use of molecular data should be considered a prerequisite for any study that requires taxonomic identification of species belonging to this genus.

In terms of underlying reasons for this host-induced morphological variation, Fryer [7] suggested that the anchor of *L. cyprinacea* is reduced, and that branching of the dorsal anchors (processes) tends to be suppressed, when it parasitizes on a small host, where a limited volume of tissue would limit their full development. However, we found that some of these specimens with suppressed dorsal anchors were attached on the abdomen and the base of the dorsal fin of the mosquitofish, where they appeared to have sufficient space to develop their anchors. Furthermore, *L. cruciata* was also reported on some lager-sized fish species, such as the rock bass *Cichla aenea* [17] and the white bass *Morone chrysops* [18]. We can therefore only speculate that a number of host characteristics may affect the morphology of the parasite; it could be the size of the host, the structure of the host’s muscle and the hardness of the tissue, nutritional substances that the parasite obtains from the host, skin (or mucus) chemistry, etc.

Mosquitofish (*Gambusia affinis* and *G. holbrooki*) are native to the southeastern USA, but now occur on every continent except Antarctica due to introduction programmes implemented since the early 20th century [43]. *Lernaea* spp. infestations of mosquitofish had been reported from Bangladesh [44], Turkey [43, 45] and China [24]. *Lernaea cyprinacea* specimens from Bangladesh [44] were found to exhibit a typical *L. cyprinacea* morphology, whereas specimens from Turkey [45] morphologically better corresponded to *L. cruciata*. Razavi et al. [46] investigated an infection on the Farsi toothcarp (*Aphanius farsicus*) and found that the collected *Lernaea* specimens morphologically best corresponded to *L. cruciata*, which led them to speculate that *L. cruciata* may have been translocated into the Maharlou Lake Basin by mosquitofish. *Lernaea gobioptera*, another species first described on the body surface of *Gobiopterus macrolepis* and mosquitofish in the delta of the Pearl River in China [24], is morphologically very similar to *L. cruciata* [24, 25, 46]. Both species share a typical trait: a single pair of branched holdfasts, and dorsal and ventral arms of the anchor of about equal size, which makes it look X-shaped. Therefore, we suggest that *L. cyprinacea* tends to develop a specific shape of anchors when it infects specific fish taxa, e.g. *Gobiopterus* sp. and mosquitofish, which results in misidentification of these specimens as different species.

In the experiment by Yashouv [12], specimens from mosquitofish infected by *Lernaea* larvae were found to be quite different morphologically from *L. cyprinacea*, so Fryer [7] suggested that these specimens look like a new species. Having reviewed the images in Fryerʼs paper, we argue that they resemble *L. cruciata*. Unfortunately, we could not access the original study of Yashouv [12], so we had to rely on Fryer’s review of it [7]. Regardless of this limitation, our experimental infection results correspond to those by Yashouv, which indicates that the observed anchor shape change of *L. cyprinacea* specimens when parasitizing on *Gambusia* was not an outlier. However, the specimen which had intermediate morphological characteristics between *L. cyprinacea* and *L. cruciata* in our study, and a *L. cyprinacea* specimen obtained from mosquitofish in the field [44], suggest that *L. cyprinacea* specimens infecting mosquitofish may exhibit three morphotypes: typical morphological features of *L. cruciata*; intermediate morphological characteristics; and typical morphological features of *L. cyprinacea.*

Although morphology still plays an important role in species description, identification and taxonomy [47], there is a growing amount of evidence, from a broad range of taxa, that morphology alone often does not provide adequate taxonomic resolution and that it often leads to erroneous conclusions in some taxa [48–55]. Intriguingly, this appears to be particularly widespread in parasitic taxa [54–62], and taxonomic artefacts caused by host-induced morphological variability have been reported in several other parasitic taxa. For example, a molecular study revealed that *Caryophyllaeus laticeps* tapeworms that parasitize breams are merely a morphotype of *Caryophyllaeus brachycollis* Janiszewska, 1953, which parasitizes other cyprinid fishes [63]. Also, morphological traits of *Isthmiophora melis* are highly variable and host-dependent, and without the support of molecular data they could easily lead to a misidentification of several apparently distinct species, or even genera [55]. Although molecular studies also have their limitations [64], owing to the wide use of molecular techniques in taxonomic studies, the number of valid taxa has changed in most major taxonomic groups during the last few decades. We expect that a major re-evaluation based on molecular data would also result in a notably reduced number of valid species in the genus *Lernaea*.

Although molecular data have been used in the identification of *Lernaea* species [10, 11, 14–16, 65, 66], previous molecular studies appear to have focused exclusively on *L. cyprinacea*, which is therefore the only species for which there are molecular data currently (October 2018) available in public databases. Although these also include a transcriptome [65], and the complete mitochondrial genome [66], identification of *L. cyprinacea* based on molecular data has been focused principally on partial sequences of two nuclear rRNA genes: *18S* and *28S* [11, 14–16]. This unavailability of molecular data for the remaining *Lernaea* species presents a major obstacle to their application as a tool for species identification [16]. Although our findings indicate that *L. cruciata* is a synonym of *L. cyprinacea*, due to this shortage of molecular data we cannot make this claim with confidence. We therefore urge scientists to sequence relevant genes of other *Lernaea* morphospecies and re-examine the status of the species currently recognized in this genus.

## Conclusions

The results of our experimental infections indicate that *L. cyprinacea* sometimes exhibits different morphological features when parasitizing on different hosts. Considering its wide host range, we suspect that this host-specific morphology of conspecific parasites has resulted in numerous taxonomic artefacts, i.e. misidentifications of morphotypes as new species, and that many of the described species of *Lernaea* are actually one and the same species, *L. cyprinacea*. This hypothesis should be tested and validated using both molecular and morphological data, as well as experimental laboratory infections.

## Supplementary information

**Additional file 1: Table S1.** All molecular sequences belonging to the genus *Lernaea* currently (December 2018) available on GenBank. Mt.gen. is a complete mitochondrial genome, sequence size is given in bp, C column indicates whether the sequence was used for our comparative analyses, and Ref. is the associated reference.

**Additional file 2: Table S2.** Sequences used for the comparative analyses in this study.

**Additional file 3: Table S3.** Sequences used for the phylogenetic analyses in this study.

**Additional file 4: Table S4.** Best-fit models for four datasets (*cox*1, *18S*, *28S* and concatenated), selected based on the Akaikeʼs information criterion using ModelFinder software.

**Additional file 5: Table S5.** The identity values between partial *cox*1, *18S* and *28S* genes obtained in this study and *Lernaea cyprinacea* homologues available on GenBank.

## Data Availability

The datasets supporting the conclusions of this article are included within the article and its additional files. The newly generated nucleotide sequences were submitted to the GenBank database under the accession numbers MH982192–MH982227.

## Abbreviations

*cox*1: cytochrome *c* oxidase subunit 1 gene
ML: maximum likelihood
BI: Bayesian inference
AIC: akaikeʼs information criterion
