# Supplementary material for: Morphology is not a reliable taxonomic tool for the genus Lernaea: molecular data and experimental infection reveal that L. cyprinacea and L. cruciata are conspecific
Source: Parasit Vectors. 2019 Dec 11;12:579. doi: 10.1186/s13071-019-3831-y (PMC6907117; doi:10.1186/s13071-019-3831-y)
Supplement: Supplementary file 1 — Additional file 1: Table S1. All molecular sequences belonging to the genus Lernaea currently (December 2018) available on GenBank. Mt.gen. is a complete mitochondrial genome, sequence size is given in bp, C column indicates whether the sequence was used for our comparative analyses, and Ref. is the associated reference. [file 13071_2019_3831_MOESM1_ESM.docx]

| Gene | ID | Length | Host | Country | C | Ref. |
| --- | --- | --- | --- | --- | --- | --- |
| Mt.gen. | KM235194 | 14656 | fish | China | √ | (1) |
| *18S* | DQ107554 | 1487 | *Hemiculter leucisculus* | China | √ | (2) |
| *18S* | DQ107555 | 1486 | *Cultrichthys erythropterus* | China | √ | (2) |
| *18S* | DQ107556 | 1487 | *Cyprinus carpio* | China | √ | (2) |
| *18S* | DQ107557 | 1486 | *Opsariichthys bidens* | China | √ | (2) |
| *18S* | KM281816 | 915 | cyprinid fish | N/A |  | N.A. |
| *18S* | KP235363 | 1485 | *Plecoglossus altivelis* | Japan | √ | N.A. |
| *18S* | KX258625 | 813 | *Carassius auratus* | Egypt |  | N.A. |
| *18S* | KY435939 | 932 | *Oreochromis mossambicus* | South Africa |  | (3) |
| *28S* | DQ107546 | 744 | *Hemiculter leucisculus* | China | √ | (2) |
| *28S* | DQ107547 | 744 | *Cultrichthys erythropterus* | China | √ | (2) |
| *28S* | DQ107548 | 744 | *Opsariichthys bidens* | China | √ | (2) |
| *28S* | KF751648 | 417 | *Lepomis gibbosus* | Romania |  | N.A. |
| *28S* | KF751649 | 417 | *Pseudorasbora parva* | Romania |  | N.A. |
| *28S* | KM281817 | 722 | cyprinid fish | N/A | √ | N.A. |
| *28S* | KP235364 | 712 | *Plecoglossus altivelis* | Japan | √ | N.A. |
| *28S* | KX258626 | 661 | *C. auratus* | Egypt |  | N.A. |
| *28S* | KX908211 | 706 | *Odontesthes bonariensis* | Argentina |  | (4) |
| *28S* | KY346866 | 710 | *C. auratus* | Australia |  | N.A. |
| *28S* | KY346867 | 713 | *C. auratus* | Australia |  | N.A. |
| *28S* | KY346868 | 706 | *C. auratus* | Australia |  | N.A. |

**Additional file 1: Table S1.** All molecular sequences belonging to the genus *Lernaea* currently (December 2018) available on GenBank. Mt.gen. is a complete mitochondrial genome, sequence size is given in bp, C column indicates whether the sequence was used for our comparative analyses, and Ref. is the associated reference.

**References**

1. Su, Y.B., Wang, L.X., Kong, S.C., Chen, L., Fang, R., 2016. Complete mitochondrial genome of *Lernaea cyprinacea* (Copepoda: Cyclopoida). Mito. DNA 27, 1503-4.
2. Song, Y., Wang, G.T., Yao, W.J., Gao, Q., Nie, P., 2008. Phylogeny of freshwater parasitic copepods in the Ergasilidae (Copepoda: Poecilostomatoida) based on 18S and 28S rDNA sequences. Parasitol. Res. 102, 299-306
3. Welicky, R.L., De Swardt, J., Gerber, R., Netherlands, E.C., Smit, N.J., 2017. Drought-associated absence of alien invasive anchorworm, *Lernaea cyprinacea* (Copepoda: Lernaeidae), is related to changes in fish health. Int J Parasitol Parasites Wildl 6, 430-8.
4. Soares, I.A., Salinas, V., Ponti, O.d., Mancini, M.A., Luque, J.L., 2018b. First molecular data for *Lernaea cyprinacea* (Copepoda: Cyclopoida) infesting *Odontesthes bonariensis*, a commercially important freshwater fish in Argentina. Rev Bras Parasitol Vet 27, 106-9.
