# Supplementary material for: Morphology is not a reliable taxonomic tool for the genus Lernaea: molecular data and experimental infection reveal that L. cyprinacea and L. cruciata are conspecific
Source: Parasit Vectors. 2019 Dec 11;12:579. doi: 10.1186/s13071-019-3831-y (PMC6907117; doi:10.1186/s13071-019-3831-y)
Supplement: Supplementary file 4 — Additional file 4: Table S4. Best-fit models for four datasets (cox1, 18S, 28S and concatenated), selected based on the Akaikeʼs information criterion using ModelFinder software. [file 13071_2019_3831_MOESM4_ESM.docx]

**Additional file 4: Table S4.** Best-fit models for four datasets (*cox1*, *18S*, *28S* and concatenated), selected based on Akaike Information Criterion using ModelFinder software.

| Method | *Cox1* | *18S* | *28S* | concatenated |
| --- | --- | --- | --- | --- |
| BI | GTR+F+G4 | HKY+F+I | GTR+F+I | HKY+F+I |
| ML | TPM2+F+G4 | TPM2+F+I | TIM3+F+I | TPM2+F+I |
